# Towards New Methodology for Cross-Validation of Clinical Evaluation Scales and Functional MRI in Psychiatry

**DOI:** 10.3390/jcm13154363

**Published:** 2024-07-25

**Authors:** Diyana Najar, Julian Dichev, Drozdstoy Stoyanov

**Affiliations:** 1 Faculty of Medicine, Medical University, 4002 Plovdiv, Bulgaria; fm17038@mu-plovdiv.bg (D.N.); 20101098@mu-plovdiv.bg (J.D.); 2 Department of Psychiatry, Medical University Plovdiv, 4000 Plovdiv, Bulgaria; 3 Research Institute & Strategic Research and Innovation Program for the Development of MU-PLOVDIV–(SRIPD-MUP), European Union-NextGenerationEU, 4002 Plovdiv, Bulgaria

**Keywords:** fMRI, neuroscience, paranoid-depressive, review, scale, translational, connectivity, task-based, rs-fMRI

## Abstract

Objective biomarkers have been a critical challenge for the field of psychiatry, where diagnostic, prognostic, and theranostic assessments are still based on subjective narratives. Psychopathology operates with idiographic knowledge and subjective evaluations incorporated into clinical assessment inventories, but is considered to be a medical discipline and, as such, uses medical intervention methods (e.g., pharmacological, ECT; rTMS; tDCS) and, therefore, is supposed to operate with the language and methods of nomothetic networks. The idiographic assessments are provisionally “quantified” into “structured clinical scales” to in some way resemble nomothetic measures. Instead of fostering data merging and integration, this approach further encapsulates the clinical psychiatric methods, as all other biological tests (molecular, neuroimaging) are performed separately, only after the clinical assessment has provided diagnosis. Translational cross-validation of clinical assessment instruments and fMRI is an attempt to address the gap. The aim of this approach is to investigate whether there exist common and specific neural circuits, which underpin differential item responses to clinical self-rating scales during fMRI sessions in patients suffering from the two main spectra of mental disorders: schizophrenia and major depression. The current status of this research program and future implications to promote the development of psychiatry as a medical discipline are discussed.

## 1. Introduction

Depression (DEP) is a common mental disorder which manifests with anhedonia, insomnia, low affect, feeling of guilt, cognitive, and social dysfunction which affect patient’s quality of life.

Schizophrenia (SCH) is a debilitating chronic mental disorder characterized by positive and negative symptoms, prone to relapses and comorbidities, cognitive decline and, therefore, poor prognosis. Both SCH and DEP are often stigmatized disorders, disorders of socioeconomic significance, and a major public health problem [1,2,3]. A science-based approach is needed in finding a strategy for treatment-resistant forms managing of both disorders [4,5].

These are some of the reasons why in the last decades many efforts have been directed towards finding more about their etiology, pathophysiology, and last but not least—a biomarker [6,7,8,9,10] eligible for diagnostic use.

The term “connectivity” includes structural, functional, and effective connectivity. Structural connectivity refers to the anatomical connections in the brain, while functional connectivity is time-dependent, focused on finding deviations of statistical independence of spatially remote events, for example, by measuring correlation. Effective connectivity is a process used to describe the causal influence that one region of the brain exerts over other brain regions [11,12,13].

The default mode network (DMN), central executive network (CEN), and salience network (SN) are large-scale networks in the human brain. While DMN’s main role is in internally focused processes, active in low-demand tasks, CEN could be considered “the opposite” of that—it has an important role in high-level cognitive functions, attention, and complex activities. The SN is the one regulating the DMN and CEN [14,15,16]. Each network has its main nodes as follows: DMN—medial prefrontal cortex (mPFC), posterior cingulate (PCC), precuneus, angular gyrus (AG), and hippocampal formation. CEN is composed of the dorsolateral prefrontal cortex (DLPFC) and the posterior parietal cortex (PPC). SN is composed of the anterior insula (AI), anterior cingulate cortex (ACC) and nodes in the amygdala, hypothalamus, ventral striatum, thalamus, and brainstem nuclei [17]. There is growing evidence of these networks’ role in the pathophysiology of mental disorders, which are studied mostly using fMRI.

Schizophrenia and depression are the two main spectra of mental disorders, making them suitable for our objective which would be to be able to differentiate them using translational cross-validation of clinical assessment tools and fMRI.

Emerging data on the importance of brain networks and connectivity in DEP is growing, focusing on four main networks—default mode network (DMN), affective network (AN), the reward network (RN), and the cognitive control network (CCN) [18]. The suggested triple network model in DEP has been studied, showing aberrant network connectivity [19,20], suggesting that patients suffering from major depression have an abnormal regulation of the switching between the internally focused (DMN) and the externally focused (CEN) network.

There are reported controversial results on brain networks activity and SCH. Some studies have revealed decreased DMN functional connectivity [21,22,23], while others have shown hyperconnectivity in the DMN [24,25]. Aberrant activity in the SN and, therefore, disrupted interactions within and across the DMN and CEN have been demonstrated in SCH patients [26,27], highlighting their importance SCH’s pathophysiology.

### 1.1. Current Road to Psychiatric Diagnoses

Current diagnostic practices in many medical fields leverage a multi-biomarker approach, enabling objective assessments and definitive diagnoses. In contrast, the field of psychiatry currently lacks a robust inventory of objective biomarkers [27], hindering the development of a more precise and data-driven diagnostic approach. This discrepancy persists despite significant advancements in neuroscience and technology. Addressing this gap is crucial for the advancement of psychiatric diagnosis and the implementation of more targeted treatment strategies.

The most widely used diagnostic instruments in psychiatry today are the Diagnostic and Statistical Manual of Mental Disorders (DSM) and the International Classification of Diseases (ICM) [28,29], which primarily rely on clinical symptom presentation for diagnosis. These systems do not currently integrate pathophysiological mechanisms or disease etiology into diagnostic criteria. Since clinical assessment is based on questionnaires and their interpretation which is dependent on the clinician’s judgement, experience, and the patients state of mind, it is difficult to make an objective decision on the correct diagnosis or treatment plan due to the above-mentioned reasons and the lack of specific biomarkers. Due to the aforementioned mental diseases complexity, a nomothetic approach, consisting of psychological, neurobiological, and psychopathological components is needed [3].

### 1.2. Translational Cross-Validation of Task-Related fMRI and PDS: A Novel Approach

Task-related fMRI (tr-fMRI) registers signals from different brain regions by measuring blood oxygen level-dependent (BOLD) changes in response to cognitive activities. In most studies, these activities are diagnostically irrelevant and cannot be incorporated into the diagnostic approach. In such cases, clinical assessment are performed before and after imaging, creating a temporal gap which could result in a difference in emotional states. There are some limited efforts reported in the past to incorporate first-person introspective narratives into fMRI paradigms [30,31].

In a novel paradigm (Stoyanov et al.), real-time ratings of patients are performed, using self-rating scales while simultaneously obtaining fMRI data [32]. The Von Zerssen Paranoid-Depressive Scale was chosen in order to contrast different nosological groups [33,34,35,36,37].

The main challenge in the field of translational neuroimaging is two-fold. On one hand, the resting state connectivity findings are more consistent and stable across studied populations [38], whereas the task-based fMRI methods and findings are very heterogenous and diverse. Those often include tasks and tests, specifically designed in laboratory control settings and have limited translation to clinical practice. On the other hand, the use of functional MRI to measure resting state connectivity disturbances in each and every patient is an economic effort with inappropriate cost to benefit ratio. One possible resolution would be to test the underlying neural circuits with clinically relevant tasks and then use them in clinical practice as externally validated assessment tools.

The aim of this scoping review is to gather and analyze existing data on the application of translational cross-validation using self-assessment scales in fMRI and effective connectivity, to assess the results of this convergent approach, to find whether specific or common neural circuits exist, which those circuits are and, therefore, to contribute to the long-standing debate in psychiatry.

## 2. Materials and Methods

### 2.1. Methods

The following bibliographic databases were searched from 2003 to May 2024: PubMed and Scopus. The electronic database search was supplemented by scanning relevant reviews.

The electronic database search was conducted in accordance with the PRISMA extension for Scoping Review (PRISMA-ScR) checklist.

### 2.2. Search Strategy

A scoping literature review was conducted in compliance with the inclusion and exclusion criteria in the electronic databases PubMed and Scopus. A total of 47 studies were identified. The following terms were used: independent component, psychiatric scale, paranoia scale, self-assessment scale, paranoid-depressive scale, clinical scale, task-based fMRI, effective connectivity, aberrant connectivity, depression, and schizophrenia. The terms were identified from titles or abstracts of articles using a Boolean operator to achieve accurate results. Search results abstracts were screened by two reviewers. In case of a disagreement, the reviewers had a thorough discussion and drew a conclusion. Full text screening followed where 14 studies were included in this scoping review.

### 2.3. Eligibility Criteria

In this scoping review, we included original studies focused on translational cross-validation of clinical assessment scales and fMRI. For this purpose, two patient groups were chosen: depression and schizophrenia patients, all of which were adults.

The inclusion criteria were as follows: (1) Articles related to task-based fMRI or effective connectivity in depression and schizophrenia; (2) articles published in the last 20 years (2004–2024); (3) articles published in English or translated to English; (4) articles published in scientific journals; (5) articles including patient groups (depression and/or schizophrenia); (6) articles including an adult population; (7) articles that were complete; (8) original studies.

The exclusion criteria were as follows: (1) Articles that were not related to task-based fMRI or effective connectivity in depression and schizophrenia; (2) articles published more than 20 years ago; (3) articles published in languages different than English or not translated to English; (4) articles published in non-scientific journals; (5) articles not including patient groups with depression and/or schizophrenia; (6) articles including a non-adult population; (7) articles that were incomplete; (8) systematic reviews.

## 3. Results

**Figure 1:**
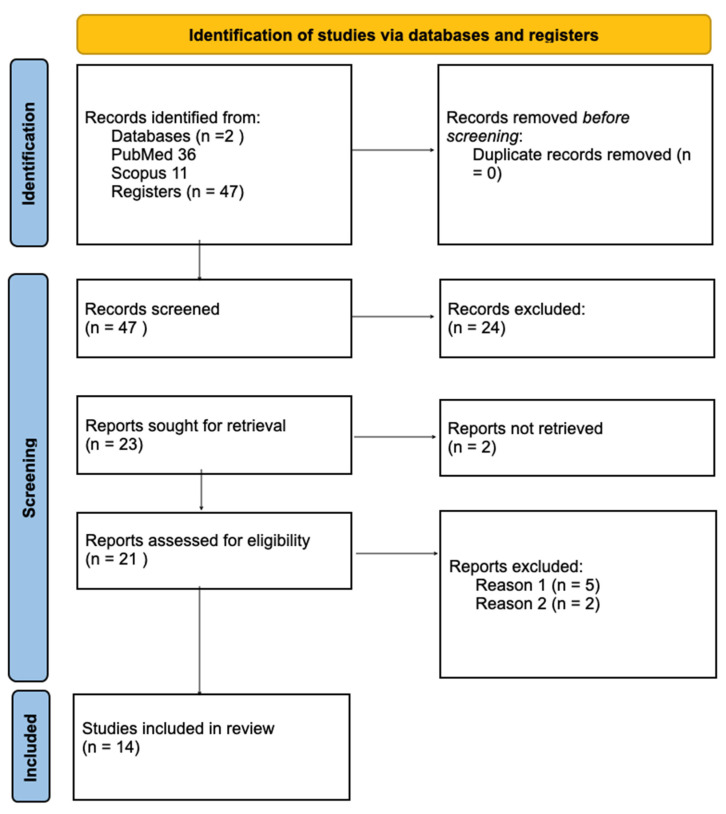
PRISMA 2020 flow diagram for new systematic reviews which included searches of databases and registers only. Reason (1): Articles that were not related to task-based fMRI in depression and schizophrenia. Reason (2): Articles that were not related to effective connectivity in depression and schizophrenia. Reason (3): Articles not including patient groups with depression and/or schizophrenia.

In Figure 1 there is provided graphical representation of the procedures for identification of the relevant studies as well as characteristics of the sources of evidence used as eligibility criteria.

### 3.1. Publication Year Distribution

Most of the studies were published in the last 10 years (*n* = 11). Only 1 paper was published earlier than 2013. The interest in this topic is growing, but research is scarce with only 2 or less publications a year since 2013.

### 3.2. Geographical Distribution of the Studies

The included studies were conducted by researchers from Bulgaria—43% of the articles (*n* = 6), China (21%; *n* = 3), 1 in California, 1 in Japan, 1 in Germany, 1 in France, and 1 in Poland (each is 7%; *n* = 1).

### 3.3. Findings

The crucial findings are described in Table 1.

## 4. Discussion

In this review, we analyzed studies focused on finding a data-driven approach in the search for a valid tool which could be used to help differentiate and build a more accurate classification of psychiatric disorders. The proposed paradigm consists of cross-validation of clinical self-assessment scales and functional MRI performed concurrently. The established correspondence between brain signatures and clinical evaluation tests can be considered crucial in the evolution of neuropsychiatric research and its potential clinical applications.

On another note, we discuss the results of rs-fMRI studies in patient populations and their potential role in the explanation of their ethiopathogenesis.

We discuss the findings of 14 articles on the application of the aforementioned model and the use of fMRI in DEP and SCH. Although there are not many, the articles on the topic could be planting a seed in the evolution of novel neuropsychiatric methods.

### 4.1. Schizophrenia

The results were variable with significant activations of the right angular gyrus, left posterior cingulate and precuneus, right transverse temporal gyrus [36], the right superior parietal lobule, angular gyrus, planum temporale, and thalamus [16]. Before commenting on these results we should add to this discussion the ICA finding of an association of paranoid-specific stimuli with frontal brain areas—disturbed sensory-motor networks and DLPFC, VLPFC, and OFC [47], while a resting state fMRI ICA indicated decreased positive network FC between both sides of the FPN and the language network and a decrease in negative network FC between the right FPN and the DAN [41].

Furthermore, IPL and DLPFC together with planum temporale (PT) and Broca’s area are a part of the heteromodal association cortex (HAC). HAC has an important role in higher cognitive functions (attention, language, memory, conscious thought) and its impairment could explain the occurrence of SCH symptoms as disorganized thoughts, auditory hallucinations, cognitive deficits, psychomotor poverty, and other typical symptoms. It has been hypothesized that the dysfunction specifically in IPL in SCH would cause a disruption of its functions-attention, language, and social cognition, while the abnormality of planum temporale could be responsible for language disorders, auditory hallucinations, reality-distortion syndrome, and disorganization syndrome [48]. Few studies have found a decrease in GMV in SCH in the HAC [49,50].

SCH patients demonstrated negative effective connectivity from the anterior precuneus (aPRC) to the lateral orbitofrontal cortex, differentiating them from HC and MDD patients [46]. In this article, Kandilarova et al. suggested that the inhibitory effect of the DMN on the OFC, OFC being a part of the reward processing system, can explain some of the negative symptoms in SCH—anhedonia, apathy, avolition, and social withdrawal [46,51].

One search result also reports data on reduced functional connectivity within DMN (in the right anterior paracingulate cortex) and SN (in the striatum) in SCH, supported by previous studies with similar findings [22,39,52,53,54], as some of them suggest possible explanations for the clinical manifestation of schizophrenia (negative, positive symptoms, altered cognition).

SCH patients were differentiated from HC using AI → AMY and AMY → SPL connections [44]. Another study showed SN dysfunction (reduced SN connectivity in the pallidum) in schizophrenia patients, causing depressed moods which disrupt patients’ quality of life [42]. All these findings sway us into focusing on the salience network (SN) and its dysconnectivity in the schizophrenia. SN’s role as a dynamic switch between the DMN and CEN is key in understanding the connection between fMRI findings and clinical manifestation. That goes on to say that a disrupted SN (ex. AI, dACC) connectivity would lead to abnormal activity of the other two networks, hence causing relevant symptoms. Stoyanov et al. [16] suggested that there is a common neural network in MDD and SCH that is disrupted, and the clinical syndrome depends on the direction of inhibition; in SCH that would be from the DLPFC (dorsolateral prefrontal cortex) to the AI (anterior insula), reflected in paranoid symptoms.

Studies on the DMN have shown variable results, but there is consistent evidence of its disrupted function in SCH [55,56]. Moreover, data from different studies over the years have shown dysfunctional activation patterns in the DMN, which are consistent with our findings, with a tendency of hyperactivation during rest and reduced deactivation during tasks [16,57,58]. This emphasizes the significance of task-performing fMRI, especially when the conducted task is related to the process of clinical diagnosis for ex. via clinical scales, with the idea of activating, or, we could say “to provoke” the engaged in the diagnostic process regions, and compare the results of both methods, which if consistent, create a combination of subjective (clinical interview) and objective (neuroimaging) data, helping the clinician to establish a more accurate diagnosis.

In support of these results on the importance of the discussed networks in schizophrenia are data from another article from our search, reporting reduced FC within DMN (in the right anterior paracingulate cortex) and SN (in the striatum) [39].

The findings regarding the amygdala (AMY) should not be ignored, since it’s role in mental disorders has been an ongoing topic in neuropsychiatric research. In SCH, there are additional data showing functional abnormalities as a decrease in activation and altered connectivity [59,60]. These changes result in symptomatic manifestations: emotional dysregulation, blunted affect, and social withdrawal.

A study on high and low suicide risk SCH patients found hyperconnectivity from PCC to MPFC and hypoconnectivity from MPFC to PCC (which only existed in HSR group) compared to HC [61]. This could be linked with the occurrence of executive function deficits, ideas of reference and cognitive bias, and their relation in a higher suicide risk in SCH patients.

One of our results showed that the combination of rs-fMRI with questions from clinical scales across different patient groups, including SCH, revealed information on symptom severity by measuring connectivity [62]. They included anhedonia models which found elements of the reward circuit of Williams, multiple DM nodes, SN, cingulo-opercular task control, frontoparietal task control, and visual networks.

Disruption in brain networks, especially the SN was a common finding in another study reporting enhanced connectivity between the SN and the right inferior temporal gyrus (ITG) and MTG (medial temporal gyrus), but negative FC between the SN and the left caudate and hyperconnectivity between the SN and the right paracentral gyrus [61]. Since the ITG and MTG have been referred to as task-negative brain regions, their hyperconnectivity with the SN (task-positive) during resting fMRI is considered abnormal and is not found in HC. The caudate is involved in salience processing, therefore, we would expect positive functional connectivity to the SN, which was not the case in SCH.

### 4.2. Depression

In depression, as in SCH, we found significant data related to brain networks and again, especially the ones constituting the triple-brain network model. A study performing task-based fMRI and rsfMRI uncovered that the connections involved in DEP are mainly the anterior insula (AI), orbitofrontal cortex, and hippocampus. Although no significant clusters of activations were found on the task-based fMRI in DEP, noteworthy is the reduced connectivity in rsfMRI from AI to the DLPFC [14], which corresponds with the results of other studies [15], and yet another one of our results showing negative FC between the SN and right ITG and MTG [45], both performing resting-state fMRI. The recurrence of these regions in different studies [14,45,63,64] expose a possible connection between the etiopathophysiology of MDD and the SN. An article on anhedonia used models that also found nodes in the SN, DMN and cingula-opercular task control network (COTCN), FPN task control network and visual networks. Data on anhedonia is extremely valuable in DEP as it is one of its main symptoms [62]. Although the SN was already discussed in this review, we should emphasize its role on emotional cognitive functions and social communication, which, when disrupted, cause symptoms seen in people suffering from this depressive disorder.

DLPFC (dorsolateral prefrontal cortex) is one of the regions constituting the central executive network. The occurrence of negative functional connectivity between AI and DLPFC during fMRI supports the hypothesis that depressive symptoms are caused by dysfunction in the triple-brain network model. CEN’s functions are mainly goal-oriented behavior, cognitive and executive tasks, and working memory, which are clearly impeded in DEP. This is supported by the already mentioned results from the study on MLM with findings of SN and CEN nodes in anhedonia models [62].

Stoyanov et al. [16] also reported self-inhibitory connection of the angular gyrus (main component of the DMN)—again giving plausible explanation for the clinical manifestation of depression.

One of the results investigating the transdiagnostic effects of electroconvulsive therapy (ECT) on brain networks in schizophrenia and major depressive disorder, found a correspondence between clinical improvement and ECT’s impact on large-scale brain networks. While the study focused on therapeutic applications, it also highlights the potential role of these networks in the underlying mechanisms of symptom presentation in these mental illnesses [43].

### 4.3. Differentiation of SCH and DEP Using fMRI and PDS

A part of our results discussed the differentiation between SCH and DEP using task-based fMRI. Key findings have been already mentioned and analyzed, but here we must highlight those relevant to distinguishing between the two mental disorders, using translational cross-validation.

By using translational methodology—the paranoid-depressive scale (PDS) and fMRI with independent component analysis (ICA), the following results were obtained.

Positive patterns in the parietal cortex, precuneus, inferior occipital cortex, thalamus, inferior cingulate gyrus, and the postcentral gyrus, corresponding to positive loading for depression scale (DS) and diagnostic neutral (DN) and negative loadings for the paranoid scale (PS) [37]. Knowing the role of each of these regions in the human brain, it is not difficult to connect these results with clinical manifestation. The parietal cortex is responsible for initial processing, integrating and interpreting sensory information, visuospatial processing, spatial orientation, and navigation. Here, we have also located the angular gyrus and the precuneus, which, as mentioned, are a major part of the DMN, whilst the posterior parietal cortex is a part of the CEN. These structures were discussed earlier. Most of the mentioned component elements participate in vision, sensations, and attention. These regions being positive in PS and DS could explain some of the symptoms in both disorders, but also be related to the triple network model. The positive patterns found in the same study in C2 corresponded to positive loading for DS and PS but not DN. The positive regions in this component were those participating in auditory functions: central operculum (auditory and language processing, motor control), superior temporal gyrus (integration of auditory information with other stimuli), and left hippocampus (verbal and episodic memory). These results can explain auditory hallucinations in SCH (which could be seen in psychotic depression), difficulties in processing auditory stimuli and disrupted memory. The last component in this study was negative only for the DS—positive patterns were found in the lingual gyrus, PRC, and insula, again corresponding to abnormal memory functions and also self-referential processing.

We mentioned the discovered activations in the right angular gyrus, left precuneus, left posterior cingulate, and right transverse temporal gyrus [35], and here we must add that those results were acquired in response to PS. Connecting these regions with cognitive functions (ex. social and conceptual processing), autobiographical memory processing, all of them leading us to a possible genesis of negative symptoms in SCH [65,66]. Dysfunctions in focal attention can be related to activations found in the AI [67]. When discussing positive symptoms and more specifically the internal and external auditory hallucinations seen in SCH, we can focus our attention on the activations found in Heschl’s gyrus for the PS—a region connected to efficient auditory and speech cues processing, as well as the inner voice (or the internal dialogue with oneself) [68]. In DEP, this article reported significant activations in the left middle temporal and right superior temporal gyrus, regions considered important in the context of connectivity in DEP, their connections to the DMN, or activity in the SN and these networks disruptions, all of which were commented on and thoroughly discussed previously in our review [62,67].

Both frontal motor/language and parietal regions are reported in both conditions [47], their involvement in SCH and DEP was discussed in detail in our review, and data from different sources confirm their importance [48,69]. SCH was differentiated in one component, located in the PFC (DLPFC, VLPFC, and OFC), while two components were modulated by the DS condition: PCC, PRC, occipital areas, and PHG; each of these areas has already been analyzed in the context of our topic earlier in the discussion.

A resume of our results can be found in Table 2.

## 5. Conclusions and Future Directions

Even a cursory analysis of our results can highlight that research on connectivity is the prevailing trend, whilst studies of task-based approaches are deficient, but promising. The results of our review reveal significant correlations between activation patterns in functionally defined brain regions and their underlying functional connectivity. This convergence suggests a potential neural signature for psychiatric disorders. These findings provide a foundation for the development of a diagnostic model that leverages objective connectivity biomarkers, alongside traditional subjective assessments, for a more comprehensive approach to psychiatric diagnosis. Such a model, aligned with the objective biomarker approach prevalent in other medical fields, could facilitate the creation of a more robust classification system for nosological entities.

Our proposal is to address the gap between classical psychiatric diagnostic methods and biological ones by endorsing the research and future implementation of translational cross-validation of clinical assessment instruments and fMRI. To substantiate these findings and enhance generalizability, further investigations employing this model with amplified sample sizes are warranted. Additionally, rigorous replication efforts and standardized protocols are crucial to ensure robustness and facilitate cross-study comparisons.

There are some challenges which this proposal is likely to face in field studies.

In the first place, such an approach should by all means consider structural alterations of the brain in patients with mental disorders. For instance, our previous studies indicate changes in the gray matter with voxel-based morphometry as well as with structural co-variance [70,71].

In order to generate appropriate and meaningful datasets, those have to be based on precisely defined patient populations in terms of their clinical presentation and objective imaging data. The functional imaging data would be predictably different in populations with or without structural brain alterations. The relevant alterations which can modify patient’s responses such as brain atrophy in most cases can be identified on an individual level and, therefore, motivate separate investigation of patient groups with or without brain atrophy.

The second issue in functional MRI studies is a lack of consistency in protocols and in patient selection. There is a reported great extent of heterogeneity of the data acquisition and processing settings and methods. Authors tend to use the methodological assumption of “anything goes” with often incompatible techniques for data analysis and interpretation. Also, it appears to be very difficult to control the impact of psychotropic medication, duration, and re-occurrence of illness, genetic, and immune variables, especially in the context of state-dependent studies. To obtain nomothetically sound results [72,73], carefully controlled studies need to be conducted.

The third problem which our proposal may encounter is the stability of the data. Future investigations should provide evidence of brain activation to specific questions across time. The reliability of patient’s response and imaging-based activation on the same measure has to be addressed with standard test–retest procedures, as applied in psychometric studies [74].

Another critical concern is to what extent a self-report format results in uniform and specific activation of defined brain regions corresponding to the current state of an individual patient’s clinical presentation. This requires the most comprehensive selection of the clinical measures, which may be adapted to functional MRI tasks. Those measures need to have demonstrated their psychometric ability to distinguish groups and states in clinical studies in order to be converted to fMRI paradigms [74].

## 6. Limitations

The study is focused on a very specific problem, pre-determined by critical methodological considerations and earlier advances in the field. This may have produced certain bias in literature selection. Moreover, literature in the field is limited, which made the authors include some titles which have less in common with the theme of the review than others. Furthermore, the heterogeneity of the data and the atlases used may have an influence on the results, leading to different regions being listed, while in fact, they are the same anatomically, confounding the outcome of the review.

The published results are based exclusively on Diagnostic and Statistical Manual (DSM) diagnosis. This may account for large scale variability both on the levels of clinical manifestation and neuroimaging findings. The modern conventional classifications do not allow proper and rigorous scientific revisions and that has been discussed as a major caveat in the field [72,73]. In addition, diagnostic variability has not been managed with the revisions of International Classification of Diseases either [75].

## Figures and Tables

**Table 1:** Significant results from the review.

Task/Test	Population (Diagnosis)	Results	Authors, Year
N-back test (0-back and 2-back)	Schizophrenic—low suicide risk (*n* = 19); high suicide risk (*n* = 14) Healthy controls (*n* = 15)	Patients showed hyperactivity in MPFC and PCC at 2-back task state compared with baseline LSR—unidirectional hyperconnectivity from PCC to MPFC, while HSR and HC have bidirectional effective connectivity between these regions HSR—hyperconnectivity from PCC to MPFC, and hypoconnectivity in the opposite direction compared to HC	Zhang et al., 2021 [38]
Resting-state fMRI; Voxel-based morphometry	Schizophrenia patients (*n* = 26) Matched healthy controls (*n* = 26)	Connectivity—SP group showed weaker connectivity in right paracingulate cortex (DMN) and putamen and palliudum (SN) VBM—lower GMC in paracingulate cluster (no correlation with reduced connectivity)	Orliac et al., 2013 [39]
Sequential finger-tapping task	Schizophrenia patients (*n* = 16) Depression patients (*n* = 16) Healthy controls (*n* = 16)	Schizophrenia—recruited adjacent areas—post- and precentral gyri, which decreased overnight Depression—decreased task-induced deactivation in DMN, overnight increase in basal ganglia and PFC, compared to decrease in the same regions in HC	Genzel et al., 2015 [40]
Resting state fMRI	MDD patients (*n* = 20) SCZ patients (*n* = 24) Matched HC (*n* = 43)	Patients—decreased both positive connectivity between the L and R frontoparietal control networks and negative connectivity between the medial visual and left control networks MDD—decreased negative connectivity between the left control and auditory networks SCZ—decreased positive connectivity between the bilateral control and language networks and decreased negative connectivity between the right control and dorsal attention networks	Wu et al., 2017 [41]
Resting state fMRI	SCZ patients (*n* = 21) Matched HC (*n* = 21)	Patients had lower functional connectivity in the lower R pallidum within the SN	Ohta et al., 2018 [42]
Task-based fMRI—Paranoid-Depressive Scale	SCZ patients (*n* = 15) DEP patients (*n* = 20), of which MDD patients (*n* = 10) and BD patients (*n* = 10)	SCZ—increased activation in right angular gyrus, left posterior cingulate and precuneus, right transverse temporal gyrus in paranoia vs. depression items (DP > DS contrast) MDD—increased activation in left middle cingulate and right superior temporal gyrus, same contrast	Stoyanov et al., 2019 [36]
Resting state fMRI before and after right-sided unilateral ECT	MDD patients (*n* = 8) SCZ patients (*n* = 8) HC (*n* = 20)	Patients—reduced CEN-DMN functional connectivity and increased CEN-SAL functional connectivity, with a trend for inverse correlation between the two MDD—matches with general patient findings SCZ—matches with general patient findings	Sambataro et al., 2019 [43]
Task-based fMRI—Paranoid-Depressive Scale	MDD patients (*n* = 14) SCZ patients (*n* = 16)	Three brain signatures with discrimination accuracy of 0.67, 0.83, 0.90 respectively	Stoyanov et al., 2019 [37]
rs-fMRI, clinical scale assessments, structural MRI	HC (*n* = 130) SCZ patients (*n* = 50) BD patients (*n* = 49) ADHD patients (*n* = 43)	Explained 65% to 90% of variance across dysregulated mood, anhedonia, and anxiety, compared to 22% without using the feature selection approach	Mellem et al., 2020 [44]
Task-based fMRI—Paranoid-Depressive Scale	SCZ patients (*n* = 25) DEP patients (*n* = 26), of which MDD patients (*n* = 10) and BD patients (*n* = 16)	SCZ—hyper-activation in left precuneus and left posterior cingulate gyrus, right superior parietal lobule and angular gyrus during processing paranoid vs. depression items Significant inhibitory influence of the MFG on the AI only in the SCH	Stoyanov et al., 2021 [16]
Resting state fMRI	101 subjects in four groups—healthy controls, SCZ patients, MDD patients, BD patients	HC vs. patient—self-regulatory properties of the AI; DLPFC and FEF communication; AI to AMY influence HC vs. SCZ—AI→AMY and AMY→SPL connections HC vs. MDD/BD—self-inhibition of the AI; MFG → FEF and HPC → FEF connections; AI and MFG to AMY influence	Kandilarova et al., 2021 [44]
rs-fMRI	SCZ patients (*n* = 29) DEP patients (*n* = 28) HC (*n* = 30)	SCZ—SN → right ITG and MTG and SN → right precentral gyrus were enhanced; SN → left caudate was decreased DEP—SN → right ITG and MTG was decreased	Huang et al., 2022 [45]
rs-fMRI	SCZ patients (*n* = 26) BD patients (*n* = 24) MDD patients (*n* = 33) HC (*n* = 21)	MDD vs. BD—inhibitory connection between left lateral OFC and left anterior PreCu vs. excitatory in BD SCZ vs. HC/MDD—negative effective connectivity anterior PRC to lateral OFC	Kandilarova et al., 2023 [46]
Task-based fMRI—Paranoid-Depressive Scale	SCZ patients (*n* = 27) DEP patients (*n* = 33)	1 (C14) component-independent of condition, 1 (C11) component shared between paranoid and depression-specific condition, 1 (C38) condition linked only to paranoid-specific condition, 2 (C22, C36) conditions only to depression-specific conditions, 1 (C12) component, linked to the diagnostically neutral condition	Stoyanov et al., 2023 [47]

**Table 2:** Summary of the findings. ↓—decreased; ↑—increased.

Common	Specific for DEP	Specific for SCH
↓ positive connectivity b/w left and right FPN control networks ↑ activations in central operculum, STG and the left hippocampus ↑ disruptions in frontal motor/language and parietal regions	↓ DMN deactivation during task ↓ negative connectivity b/w left control and auditory networks ↑ activation in the left MTG and right STG ↑ self-inhibition of the AI and AG	↑ activity in MPFC and PCC (CEN) ↓ connectivity in the DMN during rest ↓ connectivity in right paracingulate cortex and putamen ↓ positive connectivity b/w the bilateral control network and language networks ↓ negative connectivity b/w right control and dorsal attention network ↓ FC in the SN ↑ activation in right AG, left PCG, PRC, right transverse TG, lingual gyrus, Heschl’s gyrus
